# The effects of ideology and cognitive reflection on evidence gathering behavior in the political domain

**DOI:** 10.1371/journal.pone.0338088

**Published:** 2025-12-02

**Authors:** Florian Justwan, Bert Baumgaertner

**Affiliations:** Department of Politics and Philosophy, University of Idaho, Moscow, Idaho, United States of America; Wuhan University, CHINA

## Abstract

This paper explores individual-level standards of evidence in the political domain. In particular, we examine why people rely on different types of evidence in their evaluations of causal claims. Our empirical analysis is based on original survey data collected in August 2023. We conducted a demographically diverse online survey in the U.S in which we asked respondents to evaluate the effectiveness of a new policy initiative (cash bail reform). The survey offered subjects different pieces of information to evaluate the effectiveness of the intervention. Among other things, people could view: (a) The number of instances in which cities have/ have NOT been exposed to the policy intervention as well as observed societal outcomes for each case group; (b) Evaluations provided by in-group and out-group sources. Our empirical analysis reveals three major findings. First, standards of evidence vary systematically across individuals. In particular, respondents differ across two main dimensions: 1) the type of first-order/ statistical evidence they collect on a given question and 2) the type of expert testimony that they consult when assessing social cause-and-effect relationships. Second, both conservative ideology and people’s overall propensity to engage in cognitive reflection explain at least some of this variation. In particular, more liberal respondents as well as subjects with higher scores on the cognitive reflection scale exhibit a pronounced tendency to collect comprehensive statistical evidence rather than other forms of information. Third, people who score highly in cognitive refection are also more likely to refer to a broader range of external sources than their counterparts with lower reflection scores.

## Introduction

Perceptions about the efficacy of a policy are a major point of public contention [1–3]. Proponents and detractors often point to different bodies of evidence as basis for their respective conclusions. This paper investigates why people rely on different evidentiary standards when they assess the efficacy of a policy initiative, with a focus on individual-level variations in evidence gathering.

Existing behavioral research on evidentiary standards can be fruitfully divided into two kinds of contributions. Work on information *processing* studies how people reason from evidence [4–8], while work on information *seeking* studies how people build a body of evidence [9–15]. Unfortunately, the distinctions within and between these extant bodies of work tend to carve the “joints” of evidence in different places. Studies about information seeking tend to forgo fine-grained differentiations of statistical evidence as compared to studies in information processing. On the other hand, while work on information processing narrows in on certain details, such as cognitive heuristics when interpreting data presented in contingency tables, it tends to eschew categories that are of interest to the seeking literature, such as expert testimony. This variation in typologies of evidence creates barriers to transferring and generalizing insights between these areas.

We meet this challenge by supporting the empirics of our study with a framework that unifies a subset of extant distinctions into three types of *standards of evidence*. Formally, we define a standard of evidence as a threshold that causes a transition from the process of collecting information to making a choice based on that information. Importantly, this threshold is not merely about some *amount* of evidence, but a nuanced balance of different *types*. To unify the extant work on information seeking and processing for our purposes here, we will focus on three particular types that we construct: categorical, associative, and expert-based standards of evidence.

Our goal is to map people’s evidence gathering behavior in the context of a political initiative presented to them, and then investigate empirically which individual-level predictors determine what standards of evidence respondents rely on for this task. In particular, we investigate the extent to which two well-known predictors – ideology and cognitive reflection – apply to how people *gather* evidence. Our results establish that the fine-grained distinctions of “statistical data” in the information processing literature are also crucial to understanding information seeking.

### The effects of ideology on evidentiary standards

What makes the task of assessing the causal impact of policy initiatives particularly interesting is that it is not merely a matter of volume of evidence. Rather, there is likely a complex interplay between different types, especially when they point in different directions – the “data” from one study might suggest causal efficacy, while an expert’s judgment is to the contrary. There might also be “pockets” of agreement between some types, but disagreement across others. Moreover, whether a body of evidence points in several directions or in one depends on what information has been gathered in the first place, which can also influence what information is gathered next, and from whom.

Broadly, we want to better understand what different evidence types people select. Specifically, we want to understand how people gather evidence when given options that correspond to, roughly put, data and expert sources. Here we think it is worth paying attention to a distinction between first-order and higher-order evidence [16,17]. First-order evidence is information that directly supports the truth or falsity of a claim. Higher-order evidence is information that supports the relevance of a source. Note that systematic differences in first-order interpretations of the same data have been documented in the information processing literature [18,19] (a point we return to shortly). Another relevant note about the relationship between first-order and higher-order evidence is that they are not necessarily exclusive in the following sense. Some data can be first-order evidence (as in information used to forecast weather) and simultaneously be higher-order evidence that the source of the first-order evidence is relevant and perhaps even reliable (as in when we retrospectively look at the accuracy of past forecasts to assess performance). They also can come apart: by selecting a *source* of information *before* I know its first-order content, I can reveal that I have higher-order evidence about the source’s relevance. Thus, a focus on information *gathering* has the potential to tease apart (a) how people vary in what types of evidence they see as relevant from (b) how they process said evidence. As will become apparent in our study design, we can operationalize this idea by masking evidentiary content behind a query, e.g., “what does so-and-so say?”

The scholarship on information processing has made significant progress on understanding how responsive people are to first-order evidence presented to them [4,6–8]. In general, people evaluate evidence as more compelling if it is congruent with established worldviews, or as weaker if it comes from sources they distrust [5]. Taber and Lodge [4], for instance, show that respondents who favor gun control tend to rate pro-gun control arguments as stronger than otherwise identically sophisticated anti-gun control arguments.

Similarly, Kahan et al.’s [19] work on “motivated numeracy” suggests that people are more likely to correctly reason from evidence if the statistics provided to them are unrelated to issues that speak to deeply held ideological beliefs. This is an especially important finding from the information processing literature, demonstrating that we should be careful in how we characterize and subdivide the category of “data.” For example, in drawing causal inferences from 2x2 contingency tables, two common heuristics have been documented [18,19]. The first heuristic only considers the treatment group and compares the number of positive outcomes to the number of negative outcomes. This strategy is susceptible to the base rate fallacy, because it ignores the outcomes of the control group. The second heuristic compares the number of positive outcomes between both the treatment group and the control group, but ignores the number of negative outcomes across both. This strategy is susceptible to a confounder fallacy because it ignores information necessary to disentangle impact of the treatment from other possible influences. Both heuristics are only considering *partial* statistical data, whereas *full* consideration would be a comparison between the ratio of positive to negative outcomes in the treatment group to the ratio in the control group. This subtlety between partial and full consideration of data matters because they can point to different conclusions, and in turn impact decision making [19]. It is not known, however, whether similar nuances exist in people’s information seeking behaviors.

Unfortunately, however, extant delineations in the information seeking literature are too coarse-grained to detect such subtleties. Examples of common evidence typologies include: i) scientific, statistical, experiential, and expert evidence [15,20,21], ii) anecdotal versus scientific evidence [22,23], and iii) anecdotal, statistical, causal, and expert evidence [12]. None of these delineations are able to capture the differences in first-order interpretations that the information processing literature has demonstrated to matter.

Despite this shortcoming, the information seeking literature does have several relevant lessons to offer. An important lesson from this work is that people consider both statistical data and expert evidence [12]. Other contributions show that individual-level reactions to expert testimony are influenced by trust in the relevant sources [13,14] and engagement with statistical data depends at least partially on their perceived information gathering capacity [15]. In addition, numerous findings exist that a number of individual-level variables affect the *amount* of evidence people consult when they assess the efficacy of social processes. For instance, the intensity of evidence-seeking behavior has been shown to be significantly impacted by both short-lived psychological states, such as fear and anxiety [24,25], as well as a range of stable individual-level traits such as political ideology [9,26] and religiosity [10]. In particular, demand for additional evidence is generally higher among (1) liberals than conservatives [9], (2) non-religious people compared to their religious counterparts [10,11], and (3) those who perceive a greater social expectation to be knowledgeable about a particular issue compared to people who do not perceive these informational subjective norms [27].

Synthesizing the above considerations, we propose three types of evidentiary standards: categorical, associative, and expert-based. People who employ *categorical* standards of evidence rely on either data points that support a particular causal claim, or data points that oppose it. That is, those who rely on categorical standards only actively consider one type of data in their assessment of a proposed cause-and-effect relationship (e.g., they might consider the number people who received a vaccine for a particular disease and who still got sick).

To be clear, categorical standards are not necessarily a poor standard. Their appropriateness depends on context. For example, they are used in many routine day-to-day decision making, ranging from matters of taste (e.g., a bad meal can be sufficient to not return to a restaurant) to choices of information sources (e.g., a single mistake by a doctor can be sufficient to mistrust them). They are also used in technical fields, e.g., a mathematical generalization can be falsified by providing a single counterexample. However, categorical standards can be misapplied, as in the case of using only positive outcomes of a treatment to justify claims about trends, for example.

*Associative* standards make comparisons between aggregates. People relying on associative standards tend to make comparisons between multiple types of data points. For instance, they might compare the number of people who received a vaccine for a particular disease and who still got sick to the number of vaccinated individuals who remained healthy. Alternatively, they might compare infection rates between treated or untreated individuals. As we hinted towards above, associative standards can be either full or partial – depending on whether people collect all evidence necessary to calculate full conditional probabilities in the context of a proposed cause-and-effect relationship (a 2x2 contingency table) or if they rely on heuristic strategies and just compare two quantities of interest (such as the number of vaccinated and unvaccinated who stayed healthy).

Lastly, an *expert-based standard of evidence* is deference to external sources perceived to be relevant authorities. The specific identities of these actors are domain-specific [24]. However, people who employ these standards of evidence generally use outside sources as a guide to form opinions in their evaluations of causal processes. This means that they place comparatively less emphasis on first-order evidence (e.g., statistics that directly speak to the causal relationship in question). Importantly, expert-based evidentiary standards can be cognitively efficient; it is a form of outsourcing information processing. This is especially the case in contexts where outside sources have significantly more expertise and experience as compared to the information seeker. As such, expert-based standards of evidence are used in a wide range of fields, including the public health domain. For our purposes in particular, the sources that comprise expert-based standards of evidence will not include additional first-order information; they will simply present their judgment.

With these three types of evidentiary standards in hand, we now turn to existing theory to motivate our hypotheses. In particular, we are interested in how ideology and cognitive reflection influence our three types of evidentiary standards.

### The effects of ideology on evidentiary standards

From a theoretical perspective, we expect two variables to influence the type of evidentiary standards people rely on to assess cause-and-effect relationships. The first one is people’s political ideology. Previous work in psychology has established that liberals and conservatives differ substantially in their engagement with evidence and the process they adopt to evaluate socio-political claims [28,29]. Most important for our present purposes is a series of studies that found conservatives to have a lower general propensity to look for new evidence than their liberal counterparts [30]. This echoes Tullett et al. [9]: “liberalism is associated with greater data selection in both traditionally conservative and traditionally liberal regions of the country [which] suggest[s] that interest in novel data is not simply a correlate of being a political minority. In other words, there appears to be something about being liberal, rather than being a political outlier, that is associated with greater interest in novel data” (p.130). Similarly, relying on a simulated evidence-gathering game (“Beanfest”), Shook and Fazio [30] show that conservatives are less likely to sample novel stimuli than other study participants. In particular, the authors demonstrate that, “when confronted with novel objects that could be beneficial or harmful, politically conservative individuals tended to approach fewer objects than more liberal individuals” (p.997). Taken as a whole, this scholarship suggests that political conservatives in the United States consciously limit their exposure to new information relative to their liberal peers even in contexts where confirmation bias is not at play. Importantly, these findings align with a related strand of research which suggests that conservative ideology is negatively correlated with an individual’s predisposition for cognitive reflection [31,32] as well as increased need for certainty and rejection of ambiguity [29]. These core traits may motivate individuals on the right side of the political spectrum to limit their overall information intake in an attempt to quickly achieve cognitive closure.

The findings summarized above have important implications for the forms of evidence people consult when they assess proposed cause-and-effect relationships. Since conservatives tend to be less motivated to seek out new evidence than liberals, they should be more likely to settle for easily obtainable information in their assessment of proposed causal claims. In practice, this means that conservatives are likely to gravitate towards categorical forms of evidence and only consult one type of data (such as the number of cases in which a treatment coincided with a particular outcome). By extension, conservatives should also be less likely than liberals to rely on what we call fully associative forms of evidence — all of the information necessary to calculate full conditional probabilities in assessing a causal relationship. Simply put, because of their reduced desire for evidence gathering, we predict that conservatives (relative to liberals) will be more prone to generalize from a small number of cases with similar outcomes and less likely to engage in the expansive information search required to calculate all relevant conditional probabilities. Given these considerations, our first two hypotheses read as follows:

**Hypothesis 1**: Individuals with higher levels of self-reported conservatism are more likely to rely on categorical forms of evidence than individuals with lower levels of self-reported conservatism.

**Hypothesis 2**: Individuals with higher levels of self-reported conservatism are less likely to rely on fully associative forms of evidence than individuals with lower levels of self-reported conservatism.

### The effects of cognitive reflection on evidentiary standards

The second variable we predict to have a tangible effect on people’s evidentiary standards is cognitive reflection. This concept refers to people’s ability to override incorrect intuitive responses and engage in deeper, reflective thinking [33] and it has been found to be stable across time [34]. Broadly speaking, individuals with high levels of cognitive reflection tend to (1) have a heightened propensity to engage in deliberate, purposeful thinking [35], (2) make strategically optimal choices in laboratory settings [36], and (3) they are less likely to engage in various cognitive fallacies [37]. As such, cognitive reflection has been shown to be associated with successful identification of partisan fake news [38], and search for additional loan offers in the context of borrowing decisions [39].

Given their greater tendency to engage in deliberate thinking, we hypothesize that individuals with high cognitive reflection scores will seek out different types of evidence than those with lower scores on this dimension. In particular, highly reflective individuals should be particularly likely to reject categorical forms of evidence since they recognize that this type of data usually only provides an “incomplete picture” about the causal effect of a given intervention. For example, individuals with high cognitive reflection should quickly recognize that simply knowing the number of immunized people who remained healthy during a disease outbreak is an insufficient basis for assessing the vaccine’s effectiveness. Instead, these individuals should be drawn to fully associative evidentiary standards. This type of data allows people to compare the proportion of treated cases with a particular outcome to the relevant base rate, and it provides a more robust evaluative basis. On the other end of the spectrum, people with a low propensity to engage in cognitive reflection are quite prone to rely on intuitive thinking [35]. As such, they should be more likely to base their assessments of a given causal claim on readily available “statistics” (such as the number of treated cases with a particular outcome) rather than engaging in the more complex search for information that allows them to estimate and compare conditional probabilities. These considerations motivate our second set of hypotheses:

**Hypothesis 3:** Individuals with higher levels of cognitive reflection are less likely to rely on categorical forms of evidence than individuals with lower levels of cognitive reflection.

**Hypothesis 4**: Individuals with higher levels of cognitive reflection are more likely to rely on fully associative forms of evidence than individuals with lower levels of cognitive reflection.

People’s tendency to engage in cognitive reflection should also influence their willingness to rely on expert evidence. While cognitive reflection is conceptually distinct from cognitive ability [33], highly reflective individuals have been shown to perform higher on various small computational tasks [40]. As such, people who score high on this dimension tend to be more effective in tackling mathematical questions that require deliberate and careful cognitive engagement [36]. Based on these insights, we expect that individuals with high levels of cognitive reflection should be less likely to rely on experts in their evaluation of causal claims. Given their comparatively higher skill in performing computational tasks, highly reflective individuals should be more comfortable with assessing the efficacy of a given causal intervention based on first-order, statistical data. By contrast, low-reflection individuals should be more drawn to expert testimony as a way to avoid the cognitive effort required to interpret statistical data. In other words, these types of subjects should defer to experts rather than infer the validity of a causal claim based on first-order data. Based on this discussion, our fifth hypothesis reads as follows:

**Hypothesis 5**: Individuals with higher levels of cognitive reflection are less likely to rely on expert evidence than individuals with lower levels of cognitive reflection.

Finally, while we expect people with high levels of cognitive reflection to be less likely to rely on expert evidence, it is important to note that this proposed relationship is only probabilistic. In practice, some respondents with high values on cognitive reflection will still consult expert advice in their assessment of a given causal claim based on other individual-level features (such as trust in a particular expert) [15]. However, even for those respondents, cognitive reflection is likely to shape what *type* of expert testimony people rely on. In the United States’ polarized political landscape, individuals are often times relying on authorities associated with their preferred political party [41]. This preference for sources aligned with political in-groups is especially salient for topics and issues that are politically highly salient [42]. In this context, highly reflective individuals should be likely to recognize that a broader range of expert sources provides a stronger basis for the evaluation of a causal claim. As such, these types of people should be more willing to consult experts from ideological in-groups *as well as* ideological out-groups since they recognize the informational value that all forms of expert testimony can provide in the evaluation of a causal claim. In other words, individuals with a predisposition for analytic thinking should be less likely to disregard information provided by experts merely on the basis that these sources are aligned with non-preferred political parties. By contrast, subjects on the low end of the cognitive reflection continuum should be less inclined to see the importance of input from all types of external sources. As a result, these individuals should be more reliant on expert testimony from one side of the ideological spectrum. These considerations motivate the final hypothesis of this paper.

**Hypothesis 6**: Among those individuals who rely on expert evidence, those with higher levels of cognitive reflection are more likely to rely on a broader range of outside sources than individuals with lower levels of cognitive reflection.

## Materials and methods

### Data collection and sample characteristics

Our statistical analysis is based on data from an original online survey, conducted on August 29, 2023 (the start and end date of the study). Data collection proceeded in three steps. *First*, we designed a questionnaire containing a wide range of items about a respondent’s general socio-political characteristics, demographic questions, and a module that measures people’s evidence gathering behaviors (described in more detail below). *Second*, we obtained exemption for this research under category 2 at 45 CFR 46.101(b)(2) from the Institutional Review Board of the University of Idaho [Project Number: 23–137]. *Third*, we programmed our survey on an online platform (Qualtrics) and recruited a total of 645 respondents (minimum age: 18 years old) on the online platform Prolific Academic (ProA), a UK-based sample provider for academic research. ProA offers nationally representative samples in a number of different countries.

All survey respondents gave informed, written consent following a short study introduction that briefed them about the purpose of our survey and their rights as research participants. In particular, individuals read that “by completing and submitting your responses you certify that you are at least 18 years of age and agree to participate in the above described research study.”

In the main body of the questionnaire, we employed two attention check questions. The first one asked people to select “the number five with the letter ‘G’ next to it” from a long list of number-letter combinations. For the second one, people were asked to indicate their agreement with the following statement: “I swim across the Atlantic Ocean to get to work every day.” Here, answer options were strongly disagree, disagree (the two correct answers), agree, and strongly agree. Only respondents who correctly answered both questions were retained for the following statistical analysis. As we show in S1 Table our final sample (n = 583) matches population parameters in the United States with regards to sex, age, and race fairly well. In the next section, we discuss the variables that we used in order to test our theoretical expectations.

### Dependent variable measurement

In order to test our hypotheses, we presented respondents with a realistic but ultimately fictitious policy intervention in the United States. Individuals were presented with a plausible policy initiative with uncertain effects, and they were asked to collect information from a pre-determined “evidence bank” to evaluate the effectiveness of the program.

The specific intervention that was presented to respondents was cash bail reform. At the beginning of the survey, respondents first read some contextual information. In particular, we told our survey takers that “after a person is arrested, many defendants can await the beginning of their trial outside of jail. In order to do this, some cities require people to post bail (that is, money a defendant pays as a guarantee that they will show up in court at a later date). In other cities, most people can await the beginning of their trial outside of jail without having to post bail.”

Next, we gave people information on a fictitious policy initiative. Respondents were informed that “in the past five years, 100 of the 300 most populous American cities have implemented cash bail reform. These new laws eliminate the need to post bail for most offenses. In these 100 communities, most defendants can now await the beginning of their trial outside of jail without having to post bail. The remaining 200 most populated cities have NOT implemented cash bail reform. Instead, these communities still require people to post bail in order to stay outside of jail prior to their trial.”

After this introduction, subjects were told that they should now assess whether eliminating the need to post bail influences crime rates across the United States. We informed them that we would provide them with a number of different pieces of evidence that could help them with this evaluation. Survey-takers were told that they could view as many pieces of evidence as they would like. After they felt like they had enough evidence, they would be able to provide their final assessment.

The “evidence bank” presented to individuals contained 10 unique types of information. Respondents could select which evidence they wanted to retrieve. All available options were worded in such a way as to make it clear what type of evidence respondents would receive without giving away in what direction the evidence would point (see Table 1). After individuals clicked on a given type of evidence, the survey would display the corresponding information to them. Subsequently, respondents could decide if they had enough information to make a final evaluation or if they wanted to look at more evidence. Each respondent had to view at least one type of evidence before moving on. The maximum number of displayed pieces of evidence was 10. In our dataset, the average number of retrieved pieces of information is 4.31 (sd = 2.51).

**Table 1:** Available Evidence Types.

• How many cities that have implemented cash bail reform experienced increases in crime? (*Answer if selected*: 63). • How many cities that have implemented cash bail reform experienced decreases in crime? (*Answer if selected*: 37). • How many cities that still require people to post bail experienced increases in crime? (*Answer if selected*: 126). • How many cities that still require people to post bail experienced decreases in crime? (*Answer if selected*: 74). • Do Democrats think that cash bail reform influences crime rates? (*Answer if selected*: Most leading Democrats argue that cash bail reform has no influence on crime rates). • Do Republicans think that cash bail reform influences crime rates? (*Answer if selected*: Most leading Republicans argue that cash bail reform has driven up crime rates in the United States). • How many of America’s 300 most populous cities have experienced increases in crime? (*Answer if selected*: 189). • How many of America’s 300 most populous cities have experienced decreases in crime? (*Answer if selected*: 111). • Does the Center for American Progress think that cash bail reform influences crime rates? (*Answer if selected*: According to the Center for American Progress, cash bail reform has no influence on crime rates in the United States). • Does the NRA think that cash bail reform influences crime rates? (*Answer if selected*: According to the NRA, cash bail reform has driven up crime rates in the United States.).

Following our theoretical discussion, we generated three dependent variables. First, we categorized our respondents based on what type of statistical evidence they accessed in order to evaluate the effectives of cash bail reform. On this dimension, there are three main categories. Respondents are considered to rely on *categorical evidence* if they only consulted the number of cities that have (or have not) implemented cash bail reform and demanded to see how many of those cities have *either* experienced increases or decreases in crime. In other words, respondents in this category only retrieved information on *one* of the four cells presented in Table 2.

**Table 2:** First-Order Data Available to Survey Respondents.

	Cash Bail Reform	No Cash Bail Reform
Cities with Crime Increases	63	126
Cities with Crime Decreases	37	74

On the other end of the spectrum are individuals who requested all of the available first-order evidence. That is, they inquired about the number of cities with and without cash bail reform and what the crime trends were within the reformed and non-reformed communities. This means that they collected at least three of the evidence pieces from Table 2. We consider these respondents as employing “fully associative” evidentiary standards. By contrast, “partially associative” standards are held by respondents who consulted two quantities of interest from the above Table. In practice, many of those respondents prompted the database to reveal how many cities with crime increases had and had not instituted cash bail reform and how many communities with (or without) reform experienced increases and decreases in crime. Lastly, some respondents in our dataset (n = 17) did not consult any first-order data at all. Instead, these respondents (44% male, 19% Republican, 56% Democrat, 50% with bachelor’s degree or higher) exclusively relied on input from external sources. Given the small number of observations in this category, these cases were not included in the construction of our first outcome variable.

Our second dependent variable captures the degree to which respondents consult expert evidence in their evaluation of the policy. In order to create this measure, we first assessed the total number of pieces of evidence that people consulted, and we calculated the proportion of this number that constituted expert evidence. On average, about 17.3 percent of collected evidence pieces fall into this category.

Our final dependent variable will allow us to evaluate Hypothesis 6. Here, we focus on those respondents who selected some type of expert testimony (n = 276), and we categorize subjects depending on whether the sources they selected align with their preferred political party. In order to construct this variable, respondents were first asked what party they self-identify with. Answer options were Republicans, Democrats or Independent/ Other/ None. Those who selected “Independent/ Other/ None” were then asked to indicate which of the two major US parties they felt closer to. Taken as a whole, this two-step procedure provides us with information about the party preference for every respondent in our dataset. Next, individuals were coded based on whether the experts they consulted align with their preferred party. In U.S. public discourse, the NRA is often times described as a “wing of the GOP” [43], “Republican ally” [44], and “key conduit between candidates and conservative voters” [45]. As such, Democrats tend feel significantly more negative toward the NRA than Republican voters [46]. By contrast, the Center for American Progress has seen a significant amount of media coverage over the past few years in which it has been described as an “incubator for progressive ideas and governance” [47], “far-left think tank” [48], and “a major engine of Democratic policy” [49]. Given these considerations, for individuals who are closer to the Democratic Party, “Democrats” and the “Center for American Progress” are considered to be “in-group experts.” By contrast, for voters who self-identify with the Republican Party, “Republicans” and the “NRA” are treated as in-group sources. This procedure leaves us with a total of three categories: (1) respondents who only consult experts that align with their preferred political party (37.6 percent), (2) those who only consult testimony provided by the out-party (13.2 percent), and (3) those who consult testimony from both in- and out-party (49.3 percent).

Lastly, it is important to note that the goal of our paper is to understand people’s evidence-gathering behavior. We are not interested in evaluating people’s substantive conclusions for the fictitious cash bail initiative presented to them. While the information in Table 2 implies a null-effect (the proportion of cities with crime increases is identical among cities that have/ have not implemented the new policy), our statistical analysis does not seek to understand how people *reason from evidence*. Instead, our research design seeks to evaluate how people build the evidence required to make any sort of cause-and-effect evaluation.

### Independent variable measurement

Our two main independent variables are people’s self-reported political ideologies and their level of cognitive reflection. We measure ideology with a standard survey item. In particular, respondents are asked to place themselves on a 5-point ideological spectrum. In our sample, 23.9 percent of survey takers self-identified as “very liberal”, 28.6 percent as “liberal”, 24.9 percent as “moderate”, 16.6 percent as “conservative”, and 6.1 percent as “very conservative.”

In order to capture people’s levels of cognitive reflection, we rely on the widely used Cognitive Reflection Test (CRT-7) [33]. In this survey battery, respondents are asked to respond to seven different questions. Each of these questions has an intuitive (but incorrect) answer and respondents have to engage in deliberate, reflective thinking to arrive at a correct answer (such as: a man buys a pig for $60, sells it for $70, buys it back for $80, and sells it finally for $90. How much has he made? a) 0 dollars, (b) 10 dollars, (c) 20 dollars, (d) 30 dollars). All questions that are part of the CRT-7 battery are provided in the Appendix (S1 Text). In our sample 10.1 percent of respondents failed to give correct answers to any of these 7 questions. By contrast, 11.9 percent of survey-takers identified the correct response to all prompts. The average number of correct answers in our dataset is 3.5.

In the statistical analysis below, we also add a number of control variables to account for other factors driving people’s information gathering behavior. First, we account for gender. In some settings, women have been shown to have a higher propensity to engage in information-seeking behavior than men [50]. As a result, we control for whether a given survey-taker self-identifies as male (coded as 1) or female/other (coded as 0). Second, since the policy scenario presented to our survey respondents is politically quite salient, we account for whether a respondent self-identifies as a Democrat (50.6 percent), Independent (33.4 percent), or Republican (16.0 percent; the base category). Third, we control for political knowledge, defined as the extent to which individuals are informed about politics in the United States. Accounting for this variable is important, as individuals with higher levels of political knowledge are more likely to have a deeper understanding of the policy topic presented in the survey. As a result, they may feel less need to seek out additional information relative to less knowledgeable respondents. In line with previous research, this variable is based on three factual questions about U.S. politics [51]. The first item asked respondents whether the U.S. federal budget deficit – the amount by which the government’s spending exceeds the amount of money it collects – is now bigger, about the same, or smaller than it was during most of the 1990s (correct answer: bigger). The second question tests knowledge of institutional design by asking how many years constitute a full term in the U.S. Senate (correct answer: six). Lastly, all individuals were asked on which of the following four items the U.S. federal government currently spends the least: Foreign aid (the correct answer), Medicare, National defense, and Social Security. The final “political knowledge”-variable ranges from 0 (for respondents who do not answer any questions correctly) to 3 (for subjects who give correct responses to all items). Lastly, we also control for education – measured by the highest level of school or the highest degree that a given respondent has completed. Theoretically, respondents with higher levels of education should be more likely to rely on fully associative standards of evidence given that they have a higher capacity for analyzing and evaluating multiple types of data.

After completing the survey questions used to measure the variables described above, respondents were debriefed. In particular, all subjects were thanked for their participation and informed that the statistics presented in the section on cash bail reform were fictitious. Furthermore, participants were told that the purpose of this portion of the survey was to examine the types of evidence individuals consult when asked to learn about this particular policy issue.

Summary statistics for all variables in this paper can be found in Table 3.

**Table 3:** Descriptive Statistics.

Variable	Min	Max	Mean	Std. Dev.	Number of responses	Missing (incl. dk)
*Dependent Variables*						
Standard of Evidence	1	4	n/a	n/a	581	2
1: Categorical					74	
2: Partially Associative					163	
3: Fully Associative					328	
4: Expert Evidence only					16	
Proportion of Expert Evidence	0	1	0.18	0.24	582	1
Type of Expert Evidence	1	3	n/a	n/a	276	2
1: In-group sources only					104	
2: Out-group sources only					36	
3: Mixed sources					136	
*Independent Variables*						
CRT Score	0	7	3.54	2.26	583	0
Ideology	1	5	2.52	1.19	578	5
1: Very liberal					138	
2: Liberal					165	
3: Moderate					144	
4: Conservative					96	
5: Very Conservative					35	
Political Knowledge	0	3	1.86	0.85	583	0
1: 0 correct answers					34	
2: 1 correct answer					152	
3: 2 correct answers					258	
4: 3 correct answers					139	
Gender of Respondent	0	1	0.48	0.50	573	10
1: Male					276	
0: Female					297	
Education Level of Respondent	1	8	5.35	1.59	582	1
1: Less than High School					1	
2: Incomplete High School					7	
3: High School Graduate					65	
4: Some College, No Degree					139	
5: Two Year Associate’s Degree					63	
6: Four Year College or Univ. Degree					203	
7: Some Postgraduate School					15	
8: Postgraduate/ Professional Degree					89	
Party ID	1	3	n/a	n/a	583	0
1: Republican					93	
2: Democrat					295	
3: Independent					195	

### Methodological procedures

In order to test our theoretical expectations, we estimated a series of multiple regression models (multinomial logistic regressions for Models 1 and 3; linear regression for Model 2). Given the between-subjects research design, the unit of analysis in the following statistical investigations is the individual survey respondent. Below, we report p-values from two-tailed significance tests. All calculations were performed using STATA 17. Respondents with missing values on any of our variables were omitted from the analysis. While the study was not pre-registered, all materials and analytic code are publicly available on the Open Science Framework (OSF) platform:

https://osf.io/u2w5p

## Results

### Main analysis

Model 1 (see Table 4 as well as S1 File) provides a direct test of Hypotheses 1–4. Here, the dependent variable is the unordered outcome measure that captures what type of evidence people consulted to assess the effectiveness of cash bail reform in the United States. The base category in Model 1 is “partially associative standards.” In other words, it is those respondents who compared two quantities of interest from the following list: (1) reformed cities with crime increases, (2) reformed cities with crime decreases, (3) non-reformed cities with crime increases, and (4) non-reformed cities with crime decreases.

**Table 4:** Empirical results (Model 1).

*Multinomial* *Logistic Regression*	Outcome: Categorical Standard	Outcome: Fully Associative Standard
**Ideology**	0.425** (0.182) p = 0.019	−0.441** (0.133) p = 0.001
**CRT-7 Score**	−0.146* (0.074) p = 0.050	0.158** (0.049) p = 0.001
**Gender** (Male = 1)	0.497* (0.301) p = 0.098	−0.006 (0.207) p = 0.975
**Education**	−0.107 (0.093) p = 0.251	−0.005 (0.066) p = 0.936
**Political Knowledge**	−0.149 (0.195) p = 0.443	−0.043 (0.135) p = 0.750
**Democrat (relative to Rep.)**	0.619 (0.549) p = 0.259	−0.484 (0.423) p = 0.253
**Indep. (relative to Rep.)**	0.507 (0.455) p = 0.266	−0.077 (0.350) p = 0.826
**Constant**	−1.512 (1.014) p = 0.136	1.619** (0.743) p = 0.029
**Number of Observations**	549	
**Log-Likelihood**	−476.08	
**AIC**	984.16	

* = p ≤ 0.10, ** = p ≤ 0.05

According to our findings in Model 1, an individual’s political ideology is strongly associated with their evidence gathering behavior. The coefficient for this variable is statistically significant for people’s choice of categorical evidentiary standards (B = 0.425; std. error: 0.182; p = 0.02) and for fully associative evidentiary standards (B = −0.441 std. error: 0.133; p = 0.01). Substantively, these results suggest that an increase in conservative ideology is associated with *greater* reliance on categorical pieces of evidence and a *smaller* propensity to rely on all relevant statistical evidence made available to a given respondent. Taken as a whole, these findings provide support for Hypotheses 1 and 2.

Results are similar for our other main variable of interest. In Model 1, we find that people’s performance on the Cognitive Reflection Test is also strongly related to their evidence gathering behavior. More specifically, CRT-7 scores are positively related to selection of fully associate standards of evidence (B = 0.158; std. error: 0.049; p = 0.01) as well as rejection of categorical evidentiary standards (B = −0.146; std. error: 0.074; p = 0.05). Taken as a whole, these findings imply that those with a higher capacity for cognitive reflection are more likely to consult all statistical data that is made available to them. These results support Hypotheses 3 and 4.

**Fig 1:**
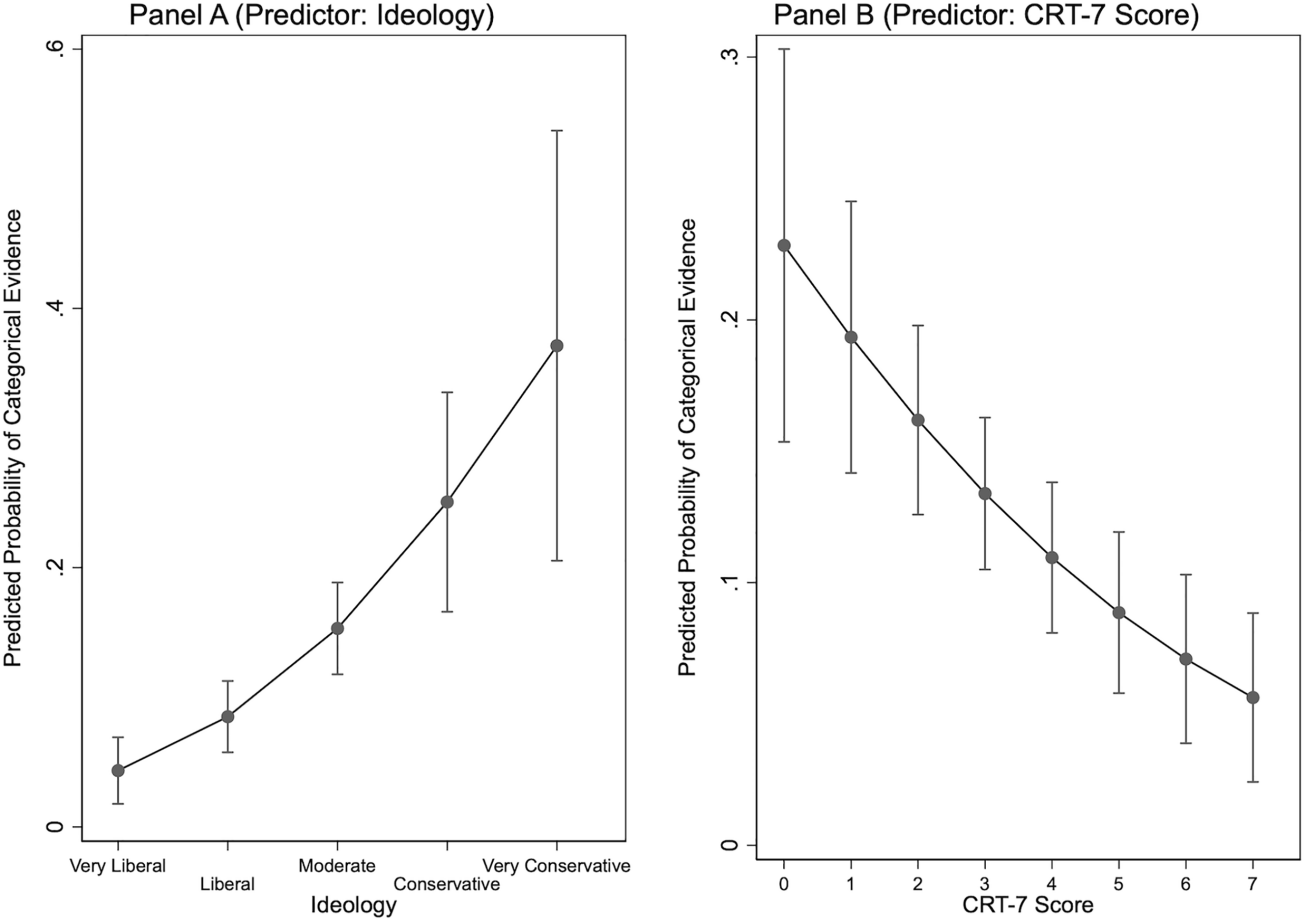
Predicted Probabilities for Categorical Standards of Evidence.

In order to visualize the substantive results from our statistical analysis, we calculated predicted probabilities. In Fig 1, we plot the probability that a given respondent relies on categorical standards of evidence (relative to “partial associative standards”, the base category). Panel A shows how these probabilities change across the full range of the ideology-variable. In Panel B, we show the same information for people’s CRT-7 scores. Holding all other variables at their observed values, a respondent who self-identifies as “very liberal” has a 4.3 percent probability of selecting a categorical form of evidence in our survey. This number then rises substantially to 15.3 percent for political moderates, and to 37.1 percent for “very conservative” respondents. Focusing on Panel B next, we see that people at the low end of the CRT-7 spectrum have a 22.8 percent probability of choosing categorical forms of evidence in the context of our cash bail reform survey. This number then decreases substantially to 5.6 percent for respondents who correctly respond to every item on the CRT-7 test.

**Fig 2:**
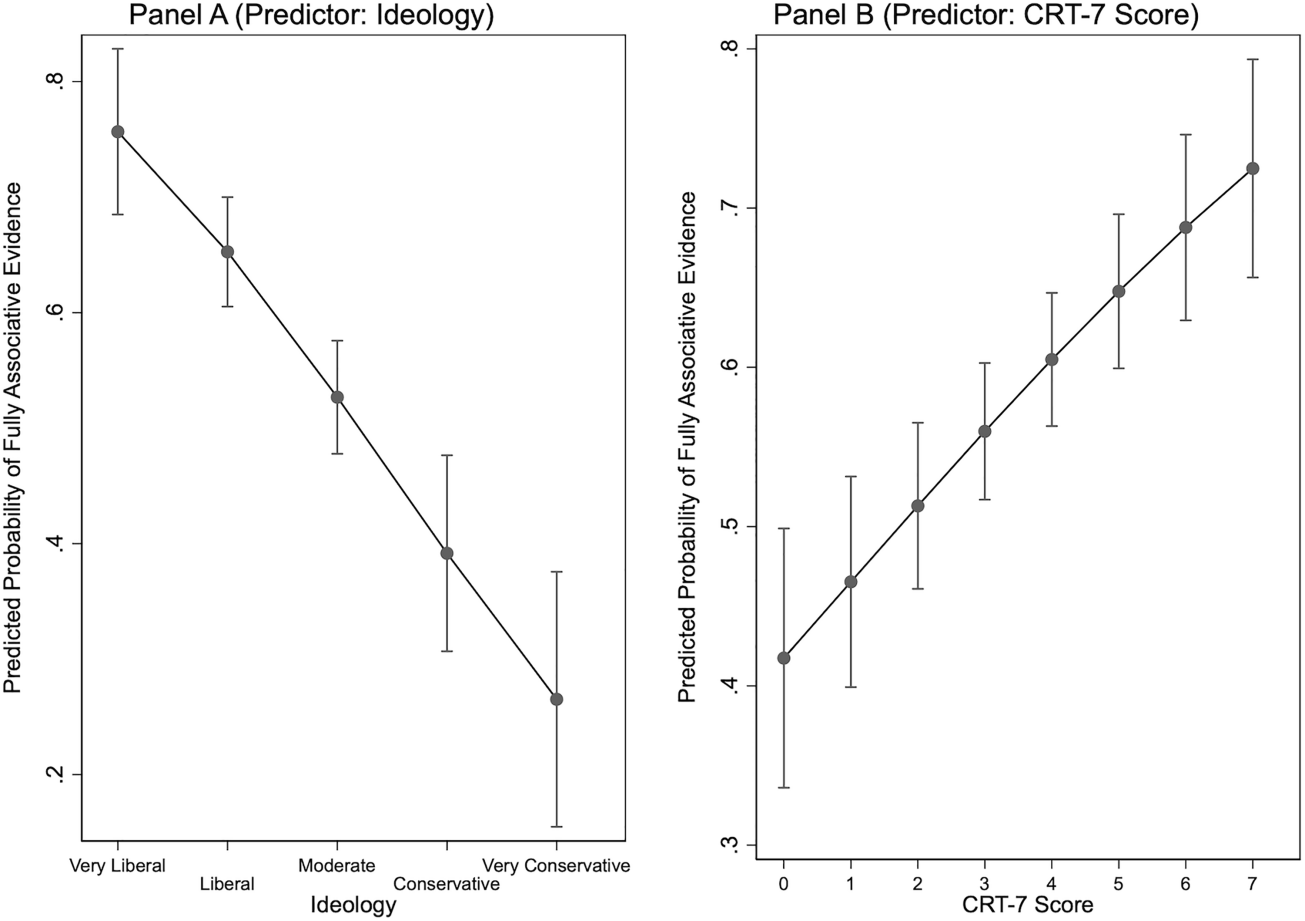
Predicted Probabilities for Fully Associative Standards of Evidence.

In Fig 2 (see below), we visualize the probability that people rely on fully associative evidentiary standards across the range of our two main independent variables. Here too, we see that the effects of ideology and cognitive reflection are not just statistically significant but also substantively important. Indeed, across the whole ideology spectrum, the probability that a given survey taker relies on all available statistical evidence goes down from 75.7 percent (very liberal respondents) to 26.5 percent (very conservative respondents). By contrast, across the full spectrum of the CRT-variable, the probability that individuals inquire about the number of cities with *and* without cash bail reform (as well as their corresponding crime trends) rises from 41.7 percent to 72.5 percent. In sum, these findings show that both ideology and cognitive reflection are important predictors for the type of evidence that people consult in their evaluation of social cause-and-effect relationships.

Next, we move to our evaluation of Hypothesis 5. In Model 2 (see Table 5 as well as S2 File), the dependent variable is the continuous measure that captures the proportion of expert evidence selected by survey respondents. In line with Hypothesis 5, Model 2 shows that people with higher scores on the Cognitive Reflection Test are significantly less likely to rely on expert testimony. The coefficient for the CRT-7 variable is negative and statistically significant (B = −0.011; std. error: 0.005; p = 0.02) which indicates that those with greater capacity for cognitive reflection tend to be more willing to consult first-order/ statistical data in their assessment of cause-and-effect relationships rather than expert assessments.

**Table 5:** Empirical results (Model 2).

*Linear Regression*	Outcome: Proportion of Chosen Expert Evidence
**Ideology**	0.014 (0.013) p = 0.285
**CRT-7 Score**	−0.011** (0.005) p = 0.022
**Gender** (Male = 1)	0.001 (0.021) p = 0.943
**Education**	−0.008 (0.006) p = 0.217
**Political Knowledge**	0.006 (0.013) p = 0.640
**Democrat (relative to Rep.)**	0.084** (0.042) p = 0.044
**Independent (relative to Rep.)**	0.027 (0.035) p = 0.449
**Constant**	0.163** (0.072) p = 0.025
**Number of Observations**	566
**R2**	0.026
**AIC**	−16.71

* = p ≤ 0.10, ** = p ≤ 0.05

**Fig 3:**
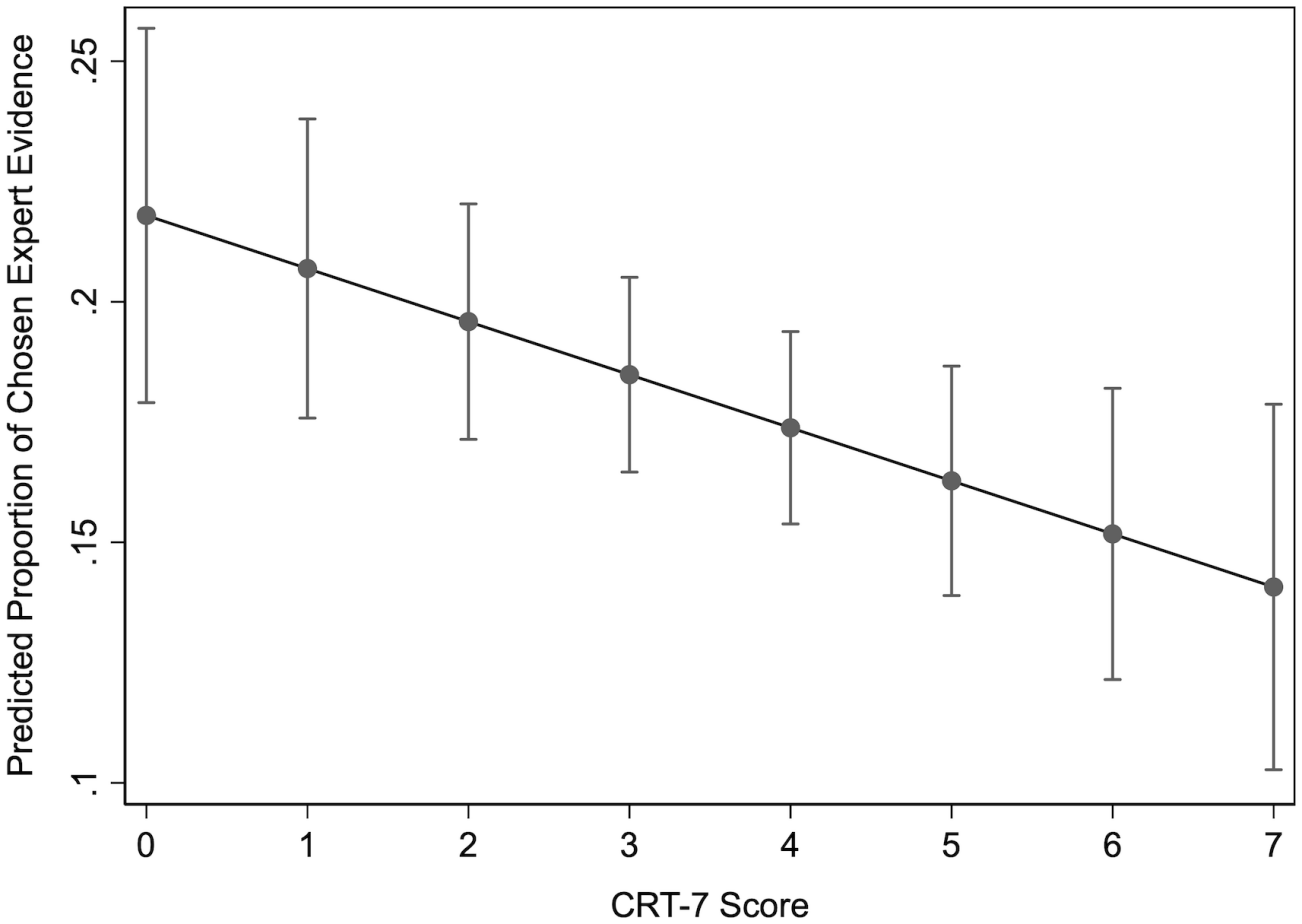
Predicted proportion of chosen expert evidence.

In Fig 3, we provide substantive effect sizes. Holding all other variables at their observed values, a respondent who receives a score of “0” on the Cognitive Reflection Test is predicted to choose expert testimony for about 21.8 percent of their evidence. However, as people’s CRT-7 scores increase, this number then goes down significantly to 14.1 percent.

Lastly, we evaluate the final testable implication of our theory. According to Hypothesis 6, people with high CRT scores should be more likely to rely on mixed expert testimony than in-group or out-group sources. In order to test this claim, we rely on a dependent variable with 3 unordered categories:(1) respondents who only consult in-group experts, (2) those who only consult out-group experts, and (3) those who consult testimony from both in- and out-party. Choosing this final category as the reference group, Model 3 (see Table 6 as well as S3 File) suggests that people’s CRT scores are indeed strong predictors of reliance on in-group and out-group experts. More specifically, our results demonstrate that respondents with high CRT scores are less likely to rely on in-group sources (B = −0.144; std. error: 0.067; p = 0.03) as well as out-group sources (B = −0.288; std. error: 0.102; p = 0.01) than mixed evidence that represents ideologically heterogenous expert testimony. This provides support for Hypothesis 6.

**Table 6:** Empirical results (Model 3).

*Multinomial* *Logistic Regression*	Outcome: In-Group Experts Only	Outcome: Out-Group Experts Only
**Ideology**	−0.004 (0.180) p = 0.981	0.133 (0.266) p = 0.617
**CRT-7 Score**	−0.144** (0.067) p = 0.032	−0.288** (0.102) p = 0.005
**Gender** (Male = 1)	−0.004 (0.291) p = 0.990	0.545 (0.430) p = 0.205
**Education**	0.071 (0.092) p = 0.438	0.150 (0.135) p = 0.268
**Political Knowledge**	0.019 (0.172) p = 0.914	−0.045 (0.256) p = 0.862
**Democrat (relative to Rep.)**	0.631 (0.655) p = 0.235	−0.850 (0.836) p = 0.309
**Indep. (relative to Rep.)**	0.203 (0.569) p = 0.722	−0.430 (0.659) p = 0.514
**Constant**	−0.591 (1.029) p = 0.565	−1.222 (1.439) p = 0.396
**Number of Observations**	260	
**Log-Likelihood**	−241.27	
**AIC**	514.54	

* = p ≤ 0.10, ** = p ≤ 0.05

**Fig 4:**
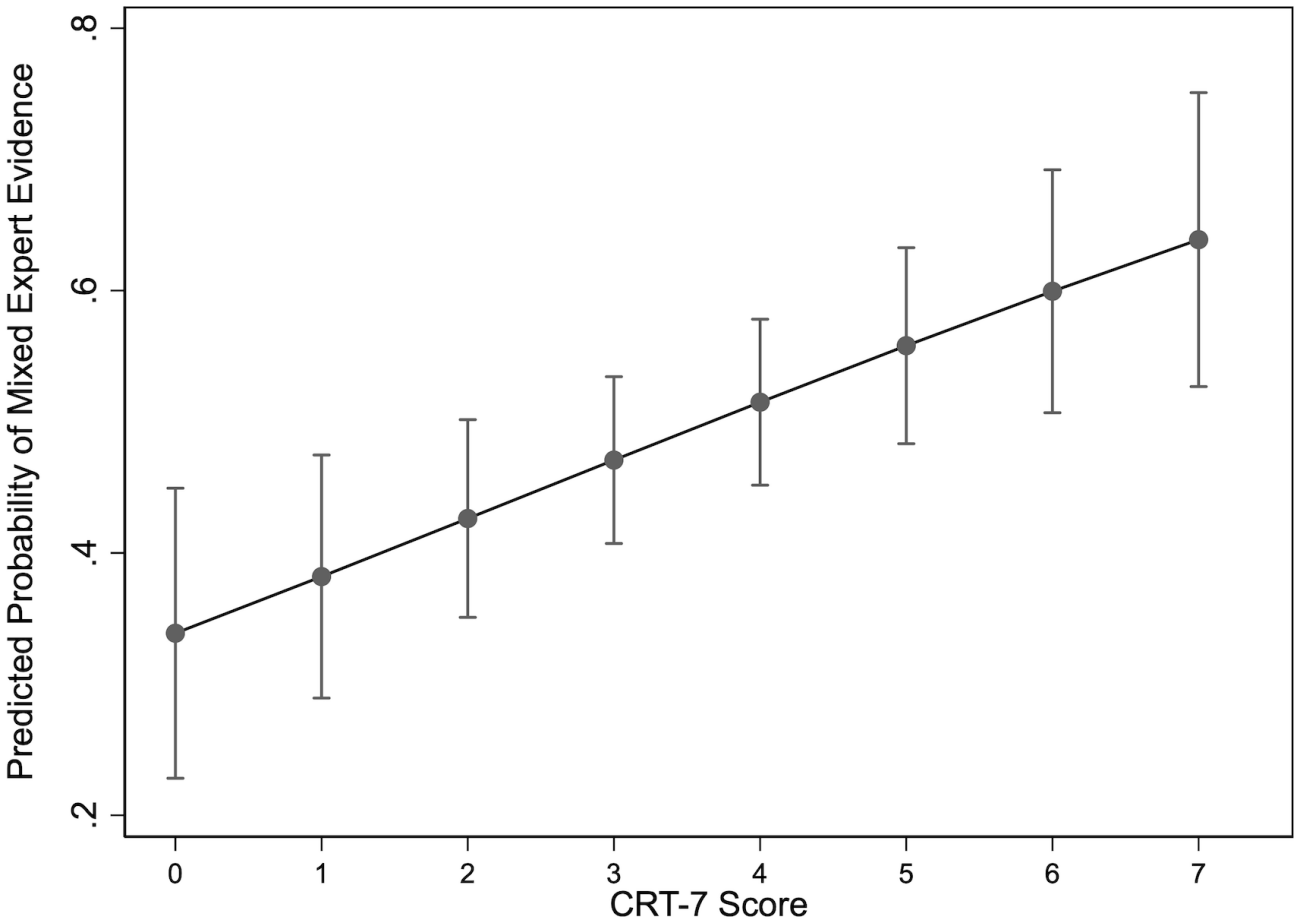
Predicted probability of mixed expert evidence.

In Fig 4, we plot the probability that a given respondent chooses mixed evidence relative to other forms of information provided in the dataset. Holding all other variables at their observed values, a survey respondent with 0 correct questions on our CRT-7 survey battery has a 33.9 percent probability of choosing a mix between in-group and out-group sources as a basis for decision-making. As people’s levels of cognitive reflection increase, the corresponding reliance on mixed evidence increases as well. In particular, at the high end of the CRT-7 spectrum, the predicted value on this final outcome variable is 63.9 percent.

### Exploratory Analysis

In a final step, we re-estimated Models 1–3, adding interaction terms between ideology and a given respondent’s CRT-7 Score. While we have not formulated any pre-theoretic hypotheses for this portion of our analysis, the following models help us to further investigate the relationships between our main variables of interest and identify avenues for future research.

Model 4 (see Table 7 as well as S4 File) is a re-estimation of Model 1. As such, the dependent variable captures what type of evidence people consulted to assess the effectiveness of cash bail reform (base category: “partially associative standards”). The regression shows that there is no conditional relationship between ideology, CRT-7 Score, and people’s propensity to choose categorical standards of evidence (B = 0.054; std. error: 0.059; p = 0.36). In other words, the effect of cognitive reflection does not vary across the range of our ideology measure. Similar results are found in the second column of Model 4. Here too, the interaction between CRT-7 Score and Ideology fails to reach statistical significance (B = 0.077; std. error: 0.040; p = 0.06). Moreover, a likelihood ratio test suggests that the inclusion of the multiplicative term does not lead to a significant improvement of fit relative to Model 1 (χ² = 3.75, df = 2, p = 0.15).

**Table 7:** Empirical results (Model 4).

*Multinomial* *Logistic Regression*	Outcome: Categorical Standard	Outcome: Fully Associative Standard
**Ideology**	0.254 (0.247) p = 0.303	−0.716** (0.199) p = 0.001
**CRT-7 Score**	−0.298 (0.197) p = 130	−0.040 (0.114) p = 0726
**IdeologyXCRT-7 Score**	0.054 (0.059) p = 0.364	0.077* (0.040) p = 0.057
**Gender** (Male = 1)	0.488 (0.300) p = 0.104	−0.025 (0.208) p = 0.904
**Education**	−0.104 (0.093) p = 0.262	−0.003 (0.066) p = 0.962
**Political Knowledge**	−0.159 (0.195) p = 0.413	−0.058 (0.135) p = 0.667
**Democrat (relative to Rep.)**	0.620 (0.546) p = 0.257	−0.452 (0.427) p = 0.290
**Indep. (relative to Rep.)**	0.489 (0.453) p = 0.280	−0.067 (0.356) p = 0.850
**Constant**	−1.092 (1.144) p = 0378	1.339** (0.845) p = 0.006
**Number of Observations**	549	
**Log-Likelihood**	−474.20	
**AIC**	984.41	

* = p ≤ 0.10, ** = p ≤ 0.05

In Model 5 (see Table 8 as well as S5 File), we focus on the variable that measures the proportion of expert evidence chosen by a particular respondent. Similar to above, we do not find evidence for a conditional relationship between our two main predictors. More specifically, the interaction term between both measures is statistically insignificant (B = 0.001; std. error: 0.004; p = 0.88) and an F-test does not allow us to reject the null hypothesis that Models 2 and 5 are identical in terms of overall fit (F[1, 557] = 0.02, p = 0.88). What this means is that individuals with higher levels of cognitive reflection are less likely to rely on expert evidence regardless of their underlying ideological orientations.

**Table 8:** Empirical results (Model 5).

*Linear Regression*	Outcome: Proportion of Chosen Expert Evidence
**Ideology**	0.012 (0.018) p = 0.527
**CRT-7 Score**	−0.012 (0.011) p = 0.250
**IdeologyXCRT-7 Score**	0.001 (0.004) p = 0.882
**Gender** (Male = 1)	0.001 (0.021) p = 0.948
**Education**	−0.008 (0.006) p = 0.219
**Political Knowledge**	0.006 (0.013) p = 0.649
**Democrat (relative to Rep.)**	0.084** (0.042) p = 0.044
**Independent (relative to Rep.)**	0.027 (0.035) p = 0.450
**Constant**	0.168** (0.081) p = 0.037
**Number of Observations**	566
**R2**	0.026
**AIC**	−14.73

* = p ≤ 0.10, ** = p ≤ 0.05

Lastly, in Model 6, we evaluate those individuals who rely on expert evidence (n = 260), and we estimate whether subjects (1) only consult in-group experts, (2) only consult out-group experts, or (3) consult testimony from both in- and out-party sources. Results can be found in Table 9 and S6 File. While there is no significant interaction between ideology and CRT-7 Score for predicting people’s reliance on out-group experts (B = −0.003; std. error: 0.082; p = 0.97), Model 6 reveals a statistically significant interaction between these two variables when predicting exclusive reliance on in-group experts (relative to consulting a mix of sources). More specifically, the multiplicative term between both variables is statistically significant (B = −0.150; std. error: 0.061; p = 0.01) and a likelihood ratio test suggests that the inclusion of the interaction improves model fit significantly (χ² = 7.03, df = 2, p = 0.03).

**Table 9:** Empirical results (Model 6).

*Multinomial* *Logistic Regression*	Outcome: In-Group Experts Only	Outcome: Out-Group Experts Only
**Ideology**	0.485* (0.270) p = 0.072	0.199 (0.253) p = 0.573
**CRT-7 Score**	0.195 (0.151) p = 0.195	−0.304 (0.253) p = 0.023
**IdeologyXCRT-7 Score**	−0.150** (0.061) p = 0.013	−0.003 (0.082) p = 0.967
**Gender** (Male = 1)	0.009 (0.294) p = 0.975	0.533 (0.430) p = 0.216
**Education**	0.077 (0.094) p = 0.412	0.151 (0.135) p = 0.216
**Political Knowledge**	0.059 (0.174) p = 0.733	−0.048 (0.258) p = 0.854
**Democrat (relative to Rep.)**	0.617 (0.689) p = 0.367	−0.871 (0.842) p = 0.301
**Indep. (relative to Rep.)**	0.267 (0.604) p = 0.659	−0.417 (0.662) p = 0.529
**Constant**	−1.864 (1.178) p = 0.114	−1.304 (1.577) p = 0.408
**Number of Observations**	260	
**Log-Likelihood**	−237.76	
**AIC**	511.52	

* = p ≤ 0.10, ** = p ≤ 0.05

**Fig 5:**
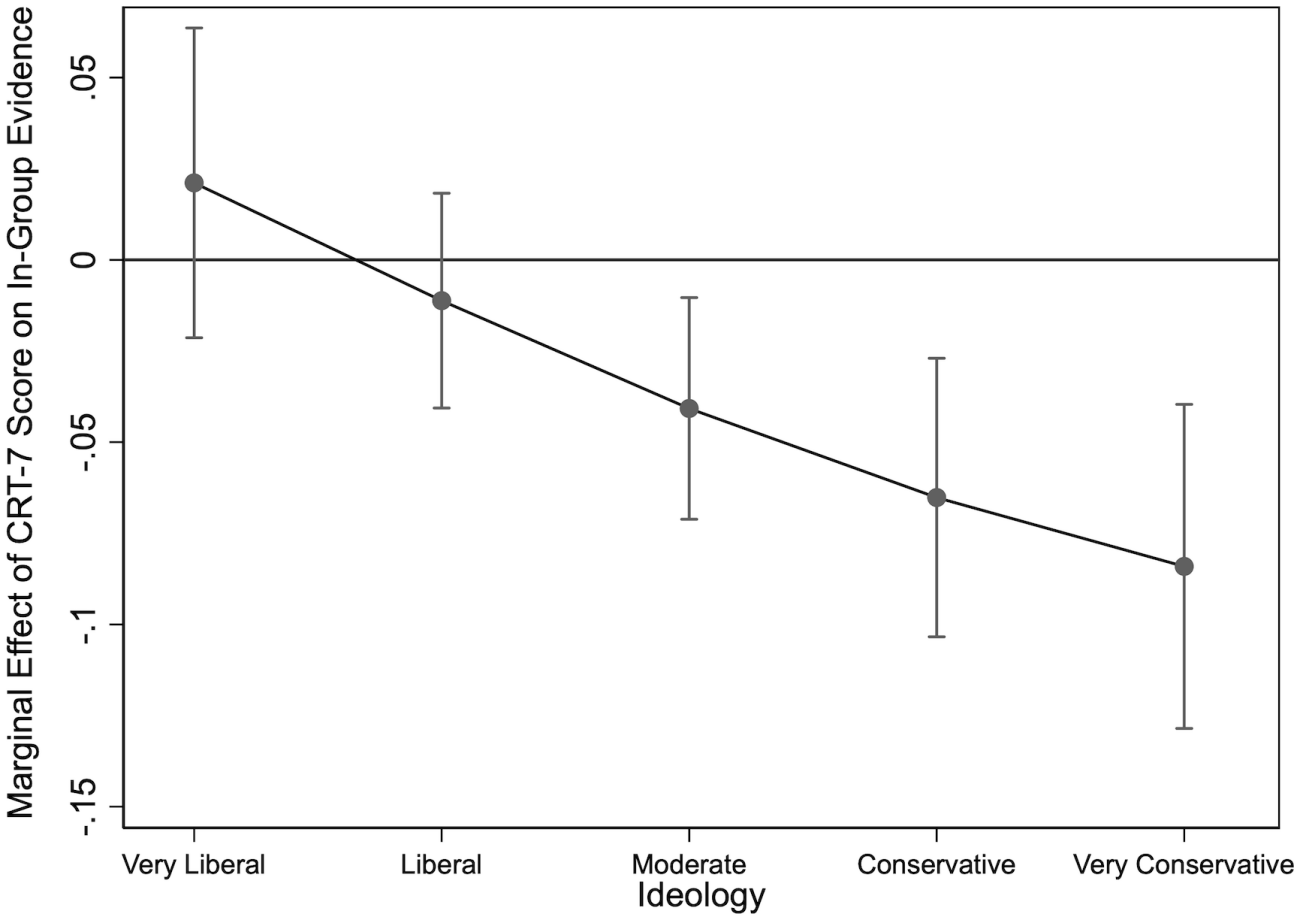
Average Marginal Effect of CRT-7 Score on In-Group Expert Evidence.

Substantive results are presented in Fig 5. Here, we plot the average marginal effect of people’s scores on the Cognitive Reflection Test on the probability that respondents only consult experts that align with their preferred political party (relative to choosing a mix of sources). The figure shows that higher cognitive reflection is associated with a significantly lower likelihood of relying on in-party sources – but only among moderates, conservatives, and strong conservatives. By contrast, individuals on the left side of the political spectrum show no significant change in their in-group source preferences as CRT-7 scores increase. Taken as a whole, this finding suggests that the relationship between cognitive reflection and source preference is not uniform across the political spectrum. An in-depth explanation for this statistical finding is beyond the scope of this paper. However, the results in Fig 5 point to a complex interplay between cognitive style and ideological orientation in predicting reliance on in-group sources. Future research should investigate the psychological mechanisms underlying this effect.

## Discussion and Conclusion

The main goal of this paper was to investigate individual-level variation in standards of evidence, especially in terms of statistical data and expert sources. In particular, we investigated why people differ in their evidentiary standards when they assess the efficacy of policy initiatives. Building on previous research in the field of psychology, our theoretical framework predicted that more conservative respondents should be (1) less likely to comprehensively consult all available statistical evidence and, instead, (2) more likely to rely on categorical forms of evidence (such as anecdotes) in their evaluation of social cause-and-effect relationships. Furthermore, we predicted that people with higher levels of cognitive reflection should be more likely to seek out *all* relevant forms of statistical evidence and less likely to rely on categorical forms of evidence. Lastly, we also expected cognitive reflection abilities to influence people’s reliance on expert sources. In particular, we predicted that those with high scores on the CRT-7 test would be less likely to rely on expert testimony in their assessment of causal claims. However, among those who do defer to third party sources, cognitive reflection should encourage people to consult a wider range of experts.

Our statistical analysis was based on original survey data, collected in August 2023. Empirically, we found broad support for our theoretical framework. In our sample, conservative ideology is associated with more reliance on categorical forms of evidence and less reliance on complete statistical information. Thus, while our findings do not speak to whether liberals and conservatives differ in how they reason from evidence (that is, what types of evidence affect their decision making), our work suggests that voters in the U.S. do differ in their engagement with first-order evidence. In other words, those on the left side of the political spectrum tend to be more likely to consult a comprehensive set of statistical data relative to those on the right. As such, our findings align with earlier work which suggests that liberals tend to have more trust in the scientific method than their conservative counterparts [52].

Beyond ideology, our results also support our predictions with regards to cognitive reflection. As expected, individuals with higher scores on the CRT-7 test are (1) less likely to rely on categorical forms of evidence, (2) more likely to seek out a full set of relevant statistics surrounding a given causal claim, and (3) less likely to defer to expert testimony overall. Furthermore, our analysis indicates that people who score highly in cognitive refection are more likely to refer to a broader range of external sources than their counterparts with lower CRT-7 scores. This suggests that cognitive reflection encourages not just deeper engagement with data, but also broader sourcing thereof.

Taken as a whole, our findings suggest that public messaging campaigns should be strategically tailored to the data collection preferences of relevant target audiences. For instance, conservative politicians likely benefit from emphasizing individual success stories or concrete, highly salient outcomes (such as the number of communities or voters who benefitted from a particular policy initiative) to their voter base. By contrast, political operatives on the left are likely to find greater success by providing broader comparative and more comprehensive data in their justifications of novel policy initiatives.

Our study has a number of limitations that provide fruitful avenues for future research. First, while our paper shows that there are systematic differences in people’s evidence gathering behaviors, it is important to note that our research design did not allow respondents to select all possible types of evidence. Most significantly, our survey did not include any evidence about other causes of crime increases/decreases in the United States. As such, survey takers were not able to learn about potential confounding variables that might be relevant pieces of information in order to assess the effectiveness of the policy intervention. Moving forward, scholars should build on our work and broaden the inquiry into the types of evidentiary standards that people adopt in the social world.

Second, in our study, respondents were asked to choose from a pre-defined list of 10 pieces of evidence. While this type of method is in line with previous research on people’s information seeking behavior [24], this somewhat static approach does not allow us to simulate the dynamic and often open-ended nature of evidence gathering in the real world. Indeed, real-world information searches (for instance, open-ended internet searches) tend to start with formulizing specific search queries which may or may not align with the content of the evidence bank employed in this study. Given these important differences, follow up research should investigate people’s engagement with evidence in a more realistic setting, giving respondents the opportunity to look for information in a free-flowing information environment.

The emergence of generative AI systems offers intriguing possibilities in this context, as they enable more adaptive and nuanced interactions with evidence that could better approximate the complexities of real-world decision-making [53]. That is, unlike traditional search engines that require users to identify relevant keywords, generative AI systems are much better at utilizing semantic similarities between questions and answers. In some respects, generative AI systems thereby better approximate how people inquire with one another, as reflected by the way that people can dialogue with chatbots much like they would with one another, e.g., “What evidence is there that...?”. That said, early work suggests that people treat AI agents differently than humans when seeking information, but how so depends on people’s own perceived information gathering capacity [54]. In short, we anticipate that generative AI will be able to provide new capabilities in studying more open-ended aspects of evidence gathering behavior.

Third, the expert entities presented to respondents in our survey varied considerably in their nature and organizational profiles. For instance, while the Center for American Progress is a policy think tank with research credentials, the NRA is primarily a political advocacy organization with a narrower and more ideologically charged profile. Likewise, both Democrats and Republicans are partisan entities rather than sources of independent policy expertise. Given these differences, it is possible that respondents perceived the overall credibility and subject matter expertise of these entities in diverse ways. Subsequent work should investigate more directly how ideology and cognitive reflection influence people’s reliance on different types of expert entities as well as what specific features of organizations make them more credible in the eyes of respondents.

Lastly, our study investigated people’s attitudes in one specific policy domain (cash bail reform). This topic is fairly politicized in the United States and supporters of both parties tend to have well-established views on the underlying issue. Given this context, it is not clear to what extent our results generalize to less polarized issues. Indeed, it is possible that the politically charged nature of the topic in question might have depressed people’s motivation to look for new information. Likewise, cognitive reflection may also function differently across domains. For example, in issue areas that touch on people’s deeply held moral beliefs, higher cognitive reflection may not facilitate higher levels of information seeking (as found in our study) but instead lead individuals to question the relevance of additional data and encourage them to rely more on value-based reasoning [55]. Thus, in order to assess the generalizability of our results, future studies should investigate people’s evidence gathering behavior in different issue contexts, including those with varying levels of political polarization or emotional investment, to determine if similar patterns hold across domains.

## Supporting information

S1 Table Sample Characteristics. (DOCX)

S1 Text Questionnaire. (DOCX)

S1 File Full Regression Results (Model 1). (DOCX)

S2 File Full Regression Results (Model 2). (DOCX)

S3 File Full Regression Results (Model 3). (DOCX)

S4 File Full Regression Results (Model 4). (DOCX)

S5 File Full Regression Results (Model 5). (DOCX)

S6 File Full Regression Results (Model 6). (DOCX)
