# Supplementary material for: The effects of ideology and cognitive reflection on evidence gathering behavior in the political domain
Source: PLoS One. 2025 Dec 2;20(12):e0338088. doi: 10.1371/journal.pone.0338088 (PMC12671747; doi:10.1371/journal.pone.0338088)
Supplement: S1 Table — (DOCX) [file pone.0338088.s001.docx]

*S1 Table: Sample Characteristics*

| **Variable** | **Population Data**  **(Census 2020)** | **Sample**  **Statistics** |
| --- | --- | --- |
| *Age Group* |  |  |
| 18-24 | 12.10% | 11.49% |
| 25-34 | 17.35% | 18.70% |
| 35-44 | 16.33% | 19.38% |
| 45-54 | 15.95% | 17.84% |
| 55 or older | 37.63% | 32.53% |
|  |  |  |
| *Sex* |  |  |
| Female | 50.93% | 51.83% |
| Male | 49.07% | 48.17% |
|  |  |  |
| *Race* |  |  |
| White | 76.91% | 78.97% |
| Asian | 6.16% | 5.86% |
| Black | 12.98 | 11.90 |
| Mixed | 2.04% | 2.06% |
| Other | 1.42% | 1.21% |
