# Supplementary material for: The effects of ideology and cognitive reflection on evidence gathering behavior in the political domain
Source: PLoS One. 2025 Dec 2;20(12):e0338088. doi: 10.1371/journal.pone.0338088 (PMC12671747; doi:10.1371/journal.pone.0338088)
Supplement: S1 Text — (DOCX) [file pone.0338088.s002.docx]

*S1 Text. Survey Questions for Variables Used In Statistical Analysis*

Gender: What is your gender?

- Male
- Female
- Non-binary / third gender
- Prefer not to say

Education: What is the highest level of school you have completed or the highest degree you have received?

- Less than high school (Grades 1-8 or no formal education)
- High school incomplete (Grades 9-11 or Grade 12 with NO diploma)
- High school graduate (Grade 12 with diploma or GED certificate)
- Some college, no degree (includes some community college)
- Two year associate degree from a college or university
- Four year college or university degree/Bachelor’s degree (e.g., BS, BA, AB)
- Some postgraduate or professional schooling, no postgraduate degree (e.g. some graduate school)
- Postgraduate or professional degree, including master’s, doctorate, medical or law degree (e.g., MA, MS, PhD, MD, JD, graduate school)

Race: Which of the following describes your race? You can select as many as apply.

- White
- Black or African-American
- American Indian or Alaska Native
- Asian or Asian-American
- Native Hawaiian or Pacific Islander
- Some other race

After a person is arrested, many defendants can await the beginning of their trial outside of jail. In order to do this, some cities require people to post bail (that is, money a defendant pays as a guarantee that they will show up in court at a later date). In other cities, most people can await the beginning of their trial outside of jail without having to post bail.

- In the past five years, 100 of the 300 most populous American cities have implemented cash bail reform. These new laws eliminate the need to post bail for most offenses. In these 100 communities, most defendants can now await the beginning of their trial outside of jail without having to post bail.
- The remaining 200 most populated cities have NOT implemented cash bail reform. Instead, these communities still require people to post bail in order to stay outside of jail prior to their trial.

We will now ask you to evaluate the effect of cash bail reform on crime rates in America's 300 most populous cities. In other words, we would like you to assess whether eliminating the need to post bail for most offenses influences crime rates in a given city.

Below, we provide you with a number of different pieces of evidence that can help you with this evaluation. You can view as many pieces of evidence as you like. After you feel like you have collected enough evidence, you will provide your final assessment.

Which of the following pieces of evidence would you like to view first?

- How many cities that have implemented cash bail reform experienced increases in crime?
- How many cities that have implemented cash bail reform experienced decreases in crime?
- How many cities that still require people to post bail experienced increases in crime?
- How many cities that still require people to post bail experienced decreases in crime?
- Do Democrats think that cash bail reform influences crime rates?
- Do Republicans think that cash bail reform influences crime rates?
- How many of America's 300 most populous cities have experienced increases in crime?
- How many of America's 300 most populous cities have experienced decreases in crime?
- Does the Center for American Progress think that cash bail reform influences crime rates?
- Does the NRA think that cash bail reform influences crime rates?

What would you like to do next?

- Look at more evidence.
- Make a final evaluation.

Note: respondents could choose up to 10 pieces of evidence.

PartyID: In politics TODAY, do you consider yourself a Republican, Democrat or independent?

- Republican
- Democrat
- Independent
- No preference
- Other Party

Partisan Lean: As of today, do you lean more to the Republican Party or more to the Democratic Party (If Respondent chose “Independent” / “No preference” / “Other party”**)**?

- Republican
- Democrat

Political Knowledge 1: Is the U.S. federal budget deficit – the amount by which the government’s spending exceeds the amount of money it collects – now bigger, about the same, or smaller than it was during most of the 1990s?

- Bigger
- About the same
- Smaller
- I don’t know

Political Knowledge 2: For how many years is a United States Senator elected – that is, how many years are there in one full term of office for a U.S. Senator?

- 1
- 2
- 3
- 4
- 5
- 6
- 7
- …
- More than 19

Political Knowledge 3: On which of the following does the U.S. federal government currently spend the least?

- Foreign aid
- Medicare
- National defense
- Social Security
- I don’t know

Attention Check 1: For quality control purposes, please select the number five with the letter "G" next to it.

- 5A
- 5B
- 5C
- G
- 5D
- 5E
- 5F
- 5G
- 5H

Ideology: In general, would you describe your political views as...

- Very liberal
- Liberal
- Moderate
- Conservative
- Very Conservative
- I don’t know

**CRT-Battery**

A bat and a ball cost $1.10 in total. The bat costs a dollar more than the ball. How much does the ball cost?

- 0 cents
- 5 cents
- 10 cents
- 15 cents
- 20 cents

If it takes 5 machines 5 minutes to make 5 widgets, how long would it take 100 machines to make 100 widgets?

- 5 minutes
- 25 minutes
- 50 minutes
- 55 minutes
- 75 minutes
- 100 minutes

In a lake, there is a patch of lilypads. Everyday, the patch doubles in size. If it takes 48 days for the patch to cover the entire lake, how long would it take for the patch to cover half of the lake?

- 24 days
- 27 days
- 37 days
- 44 days
- 47 days

If John can drink one barrel of water in 6 days, and Mary can drink one barrel of water in 12 days, how long would it take them to drink one barrel of water together?

Answer options: 1 day – 12 days.

Jerry received both the 15th highest and the 15th lowest mark in the class. How many students are in the class?

- 28
- 29
- 30
- 31

A man buys a pig for $60, sells it for $70, buys it back for $80, and sells it finally for $90. How much has he made?

- 0 Dollars
- 10 Dollars
- 20 Dollars
- 30 Dollars

Simon decided to invest $8,000 in the stock market one day early in 2008. Six months after he invested, on July 17, the stocks he had purchased were down 50%. Fortunately for Simon, from July 17 to October 17, the stocks he had purchased went up 75%. At this point, Simon has:

- broken even in the stock market
- is ahead of where he began
- has lost money

Attention Check 2: Please indicate your agreement with the following statement: I swim across the Atlantic Ocean to get to work every day.

- Strongly disagree
- Disagree
- Agree
- Strongly agree
