# Supplementary figures and images for: The effects of ideology and cognitive reflection on evidence gathering behavior in the political domain
Source: PLoS One. 2025 Dec 2;20(12):e0338088. doi: 10.1371/journal.pone.0338088 (PMC12671747; doi:10.1371/journal.pone.0338088)

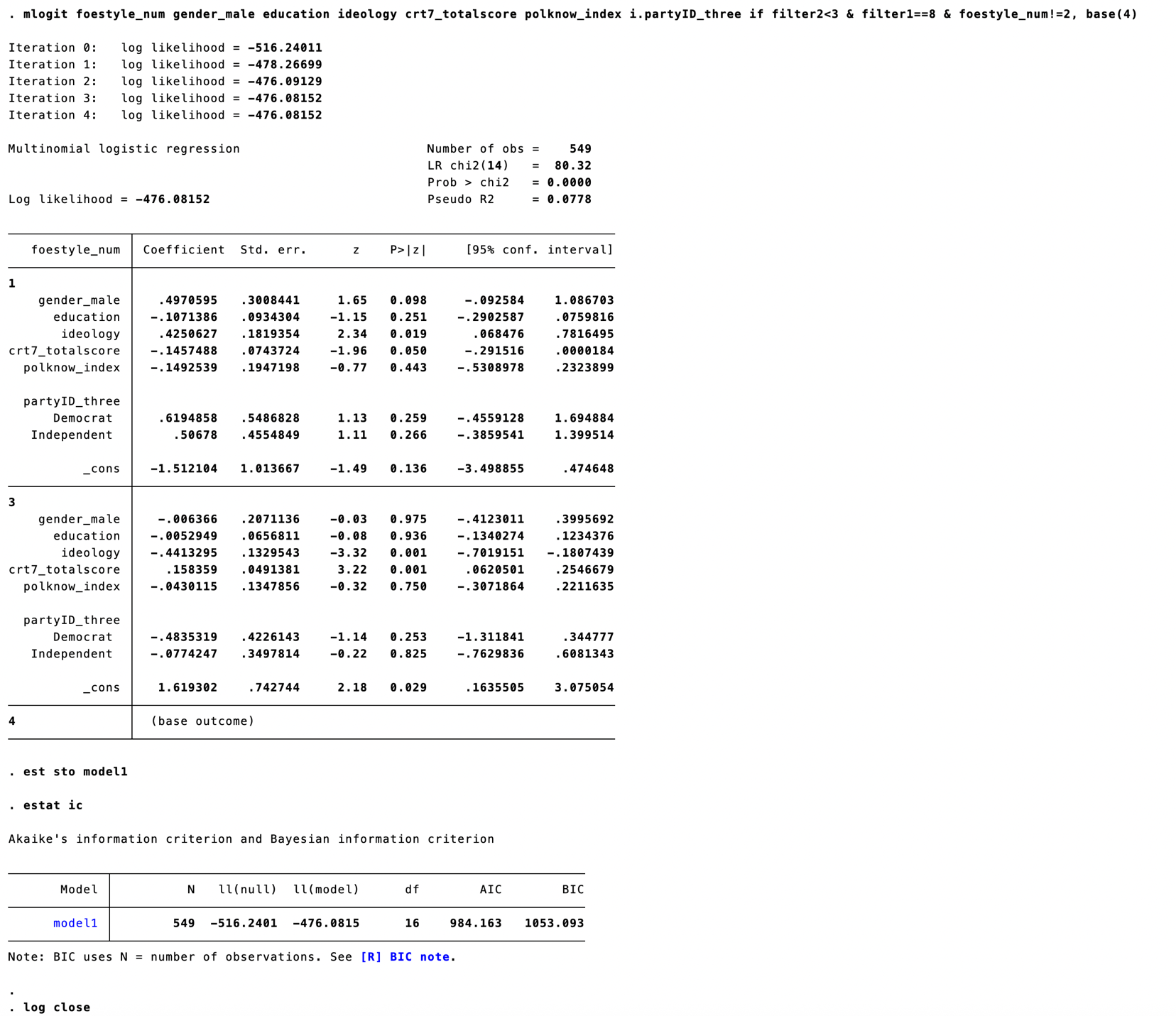

Supplement: S1 File — (DOCX) [file pone.0338088.s003.docx]

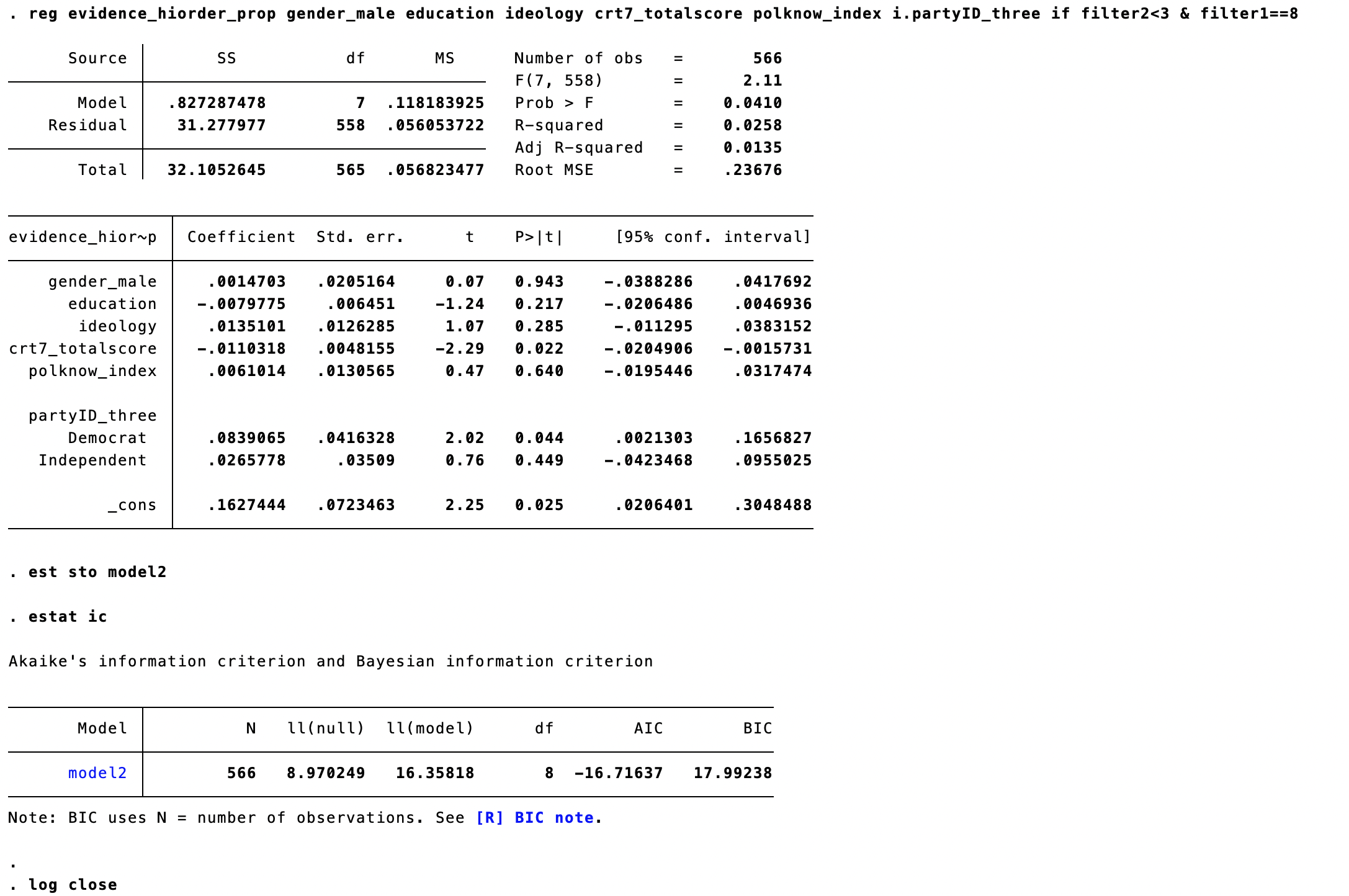

Supplement: S2 File — (DOCX) [file pone.0338088.s004.docx]

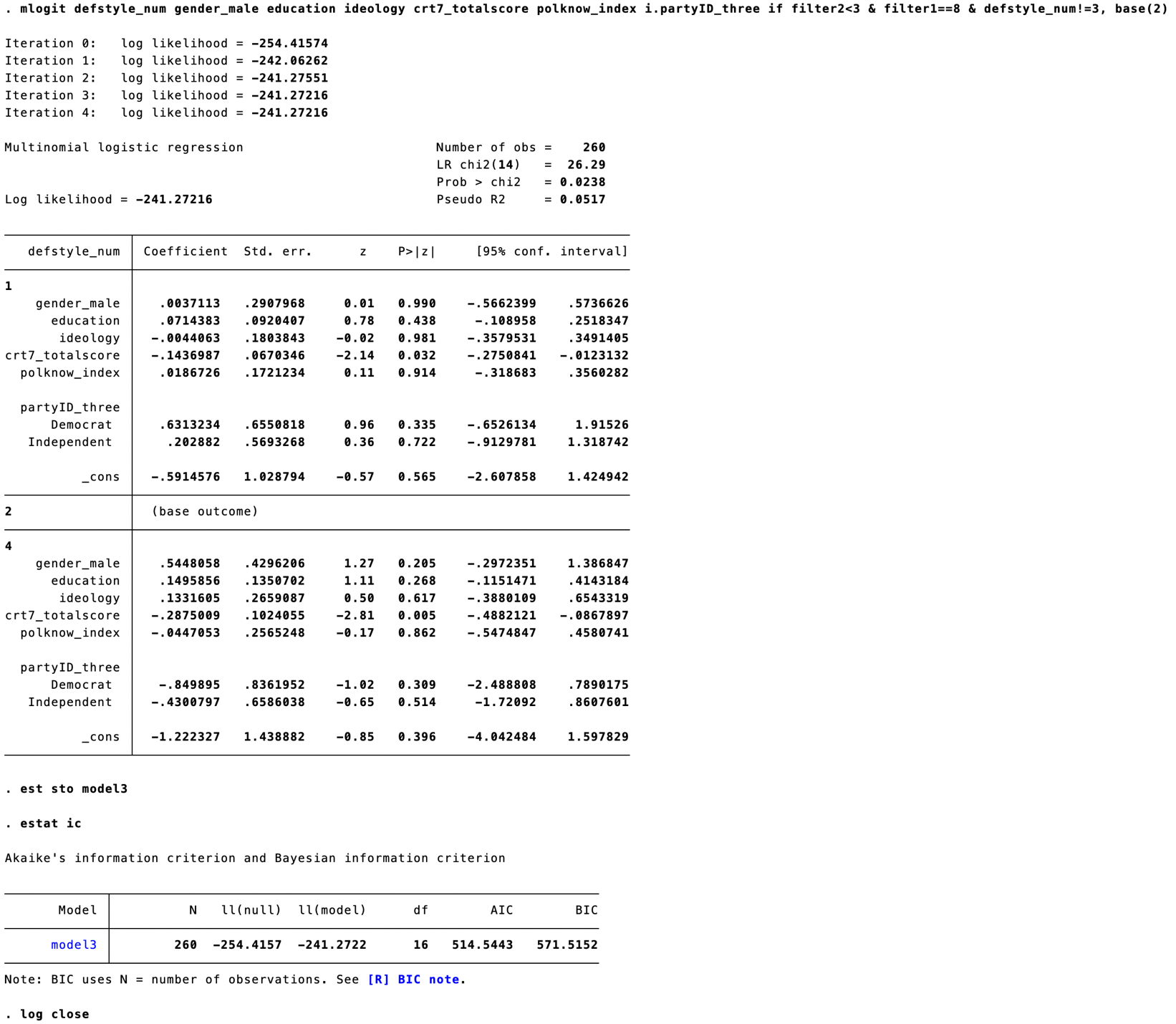

Supplement: S3 File — (DOCX) [file pone.0338088.s005.docx]

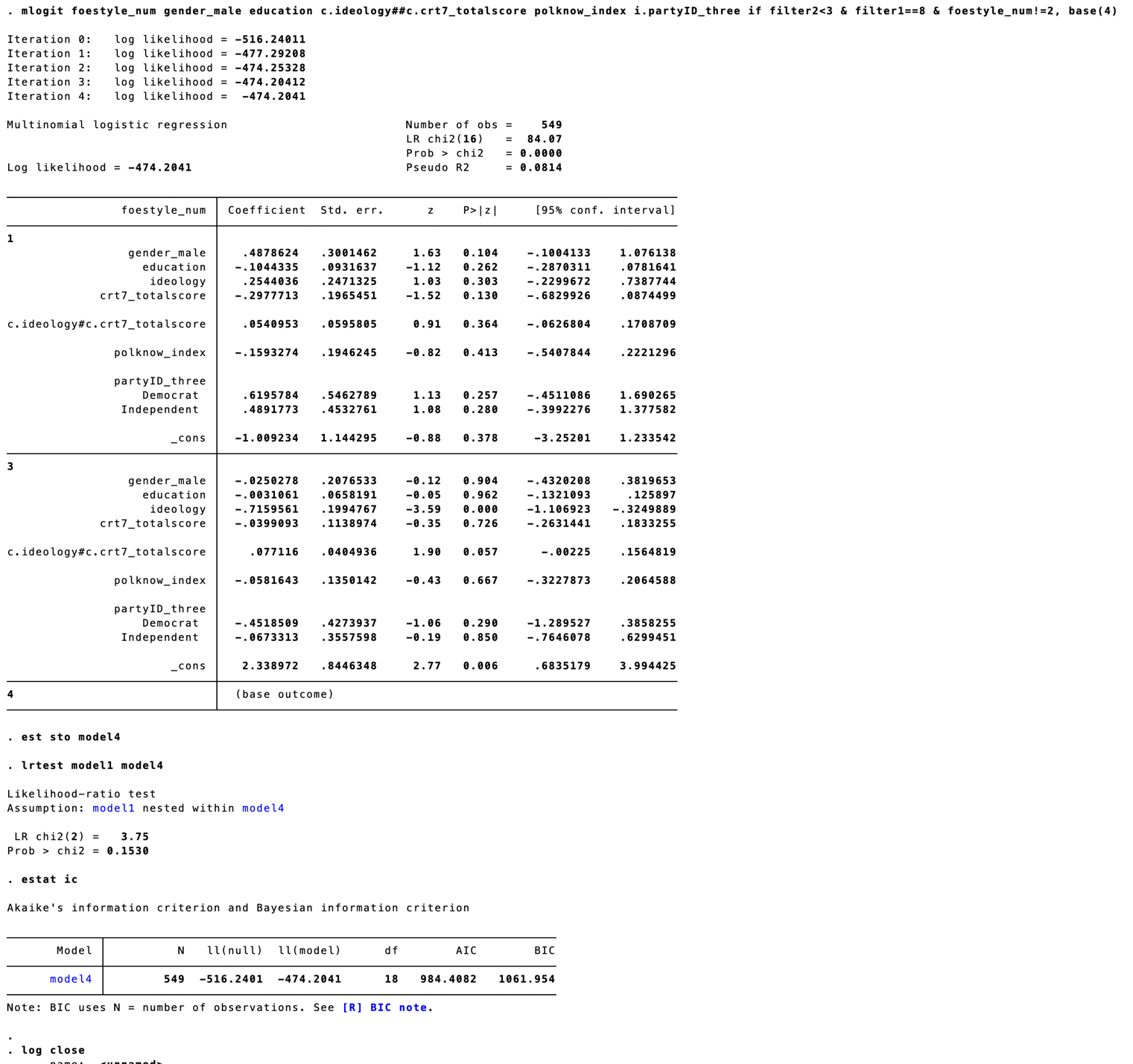

Supplement: S4 File — (DOCX) [file pone.0338088.s006.docx]

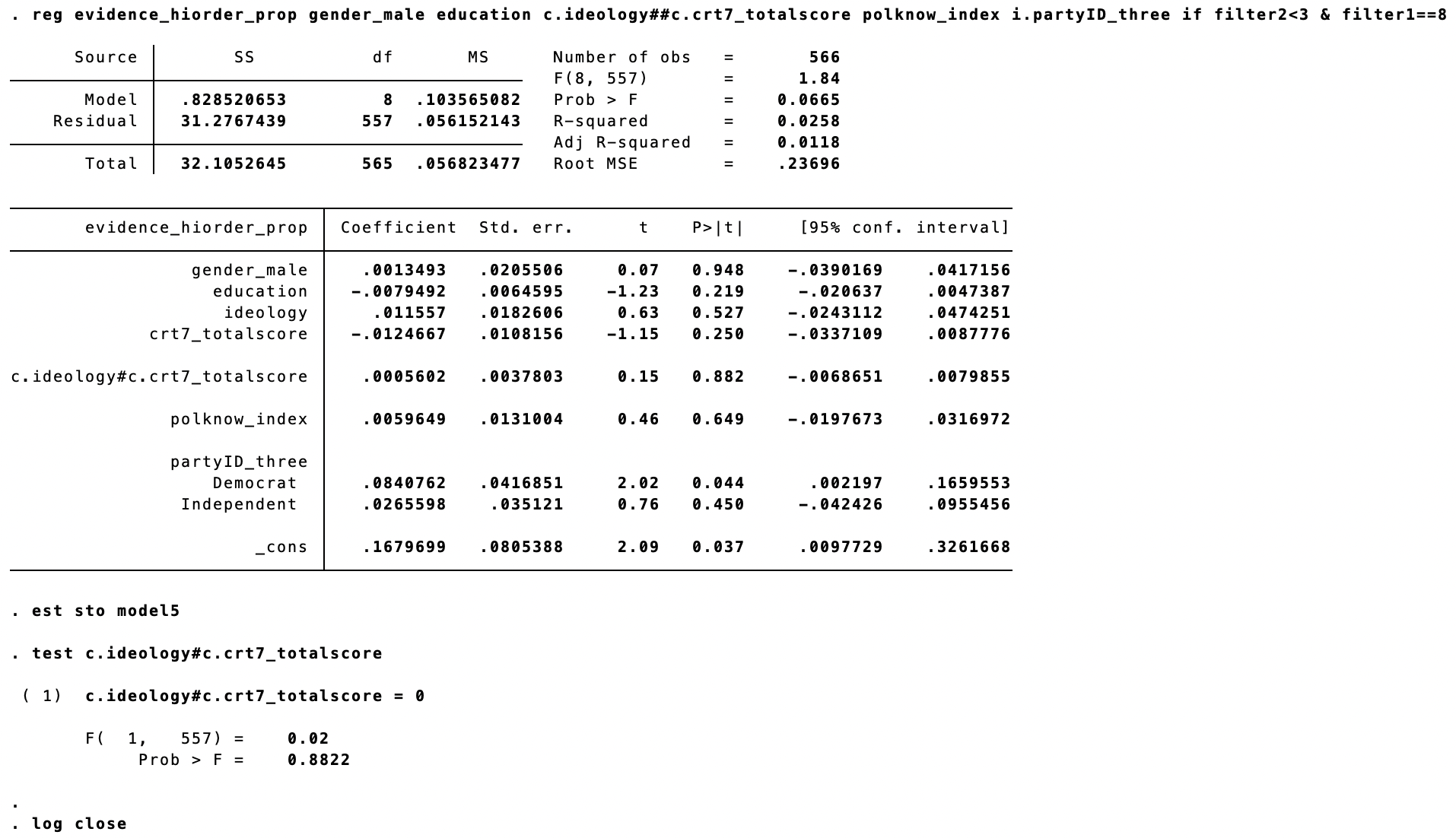

Supplement: S5 File — (DOCX) [file pone.0338088.s007.docx]

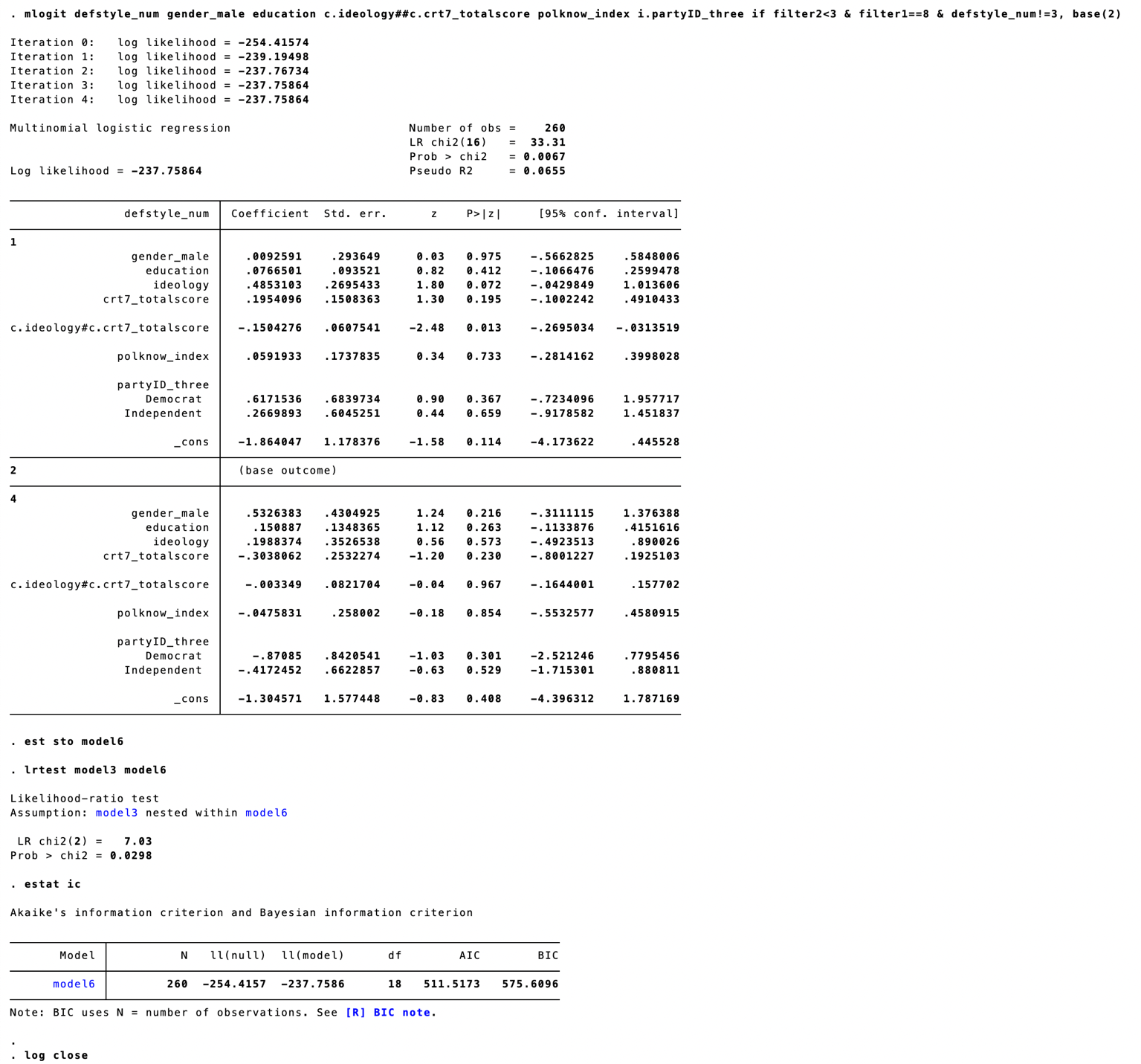

Supplement: S6 File — (DOCX) [file pone.0338088.s008.docx]
